# Protocol for the process evaluation of interventions combining performance-based financing with health equity in Burkina Faso

**DOI:** 10.1186/s13012-014-0149-1

**Published:** 2014-10-12

**Authors:** Valéry Ridde, Anne-Marie Turcotte-Tremblay, Aurélia Souares, Julia Lohmann, David Zombré, Jean Louis Koulidiati, Maurice Yaogo, Hervé Hien, Matthew Hunt, Sylvie Zongo, Manuela De Allegri

**Affiliations:** University of Montreal Hospital Research Center (CRCHUM), 850 Saint-Denis, 3rd Floor, Montréal, QC H2X 0A9 Canada; University of Montreal School of Public Health, 7101 Avenue du Parc, 3rd Floor, Montréal, QC H3N 1X9 Canada; Institute of Public Health, Medical Faculty, Heidelberg University, Im Neuenheimer Feld 324, 69120 Heidelberg, Germany; AFRICSanté & Université Catholique de l’Afrique de l’Ouest - Unité Universitaire de Bobo-Dioulasso, 01 BP 298 Bobo-Dioulasso, Burkina Faso; Centre MURAZ, 01 BP Bobo-Dioulasso, Burkina Faso; Institut de recherche en sciences de la santé (IRSS) du CNRST, 03 BP 7192 03 Ouagadougou, Burkina Faso; School of Physical and Occupational Therapy, McGill University, 3630 Promenade Sir William Osler, 2nd Floor, Montréal, QC H3G 1Y5 Canada; Institut des Sciences des Sociétés (INSS-CNRST), 03 BP 7047 Ouagadougou, Burkina Faso

**Keywords:** Performance-based financing, Community-based health insurance, User fee exemption, Equity, Burkina Faso, Implementation study, Process evaluation, Case study methodology, Research protocol

## Abstract

**Background:**

The low quality of healthcare and the presence of user fees in Burkina Faso contribute to low utilization of healthcare and elevated levels of mortality. To improve access to high-quality healthcare and equity, national authorities are testing different intervention arms that combine performance-based financing with community-based health insurance and pro-poor targeting. There is a need to evaluate the implementation of these unique approaches. We developed a research protocol to analyze the conditions that led to the emergence of these intervention arms, the fidelity between the activities initially planned and those conducted, the implementation and adaptation processes, the sustainability of the interventions, the possibilities for scaling them up, and their ethical implications.

**Methods/Design:**

The study adopts a longitudinal multiple case study design with several embedded levels of analyses. To represent the diversity of contexts where the intervention arms are carried out, we will select three districts. Within districts, we will select both primary healthcare centers (*n* =18) representing different intervention arms and the district or regional hospital (*n* =3). We will select contrasted cases in relation to their initial performance (good, fair, poor). Over a period of 18 months, we will use quantitative and qualitative data collection and analytical tools to study these cases including in-depth interviews, participatory observation, research diaries, and questionnaires. We will give more weight to qualitative methods compared to quantitative methods.

**Discussion:**

Performance-based financing is expanding rapidly across low- and middle-income countries. The results of this study will enable researchers and decision makers to gain a better understanding of the factors that can influence the implementation and the sustainability of complex interventions aiming to increase healthcare quality as well as equity.

## Background

Universal health coverage entails that everyone should have access to good quality healthcare services without experiencing financial hardship resulting from healthcare payment [1]. Despite major healthcare reforms in many low- and middle-income countries (LMICs), access to quality healthcare remains inadequate. On average, in LMICs, the maternal mortality ratio is 230 for 100,000 live births and the mortality ratio for children under the age of 5 is 53 per 1,000 [2,3]. Meager improvements have led to heated international debates on the best policies to improve access to quality healthcare services and reduce health inequity.

Recently, numerous LMICs have introduced performance-based financing (PBF) to improve the delivery of healthcare services. The World Bank, for instance, supported the design and implementation of PBF in over 30 LMICs [4,5]. Different PBF labels exist and are associated with different types of incentives and payment arrangements [6,7]. PBF generally entails that performance agreements between Ministries of Health and healthcare facilities are established to define indicators and targets to be reached in delivering these services. According to the model proposed by the World Bank, bonus payments to health providers are made after verification by reviewers of the quantity and quality of healthcare services. Across settings, facilities retain different degrees of autonomy in deciding how to use PBF payments. The underlying assumption of PBF is that financial incentives, combined with increased supervision and enhanced autonomy, will motivate health providers to improve the quantity and quality of services delivered [8].

The body of evidence on the implementation and effectiveness of PBF is not well established. In Rwanda, for instance, PBF increased the number of assisted deliveries, children's consultations, and perceived quality of care [9,10]. Meanwhile, in Democratic Republic of Congo, PBF resulted in lower out-of-pocket payments for patients while receiving equal or better quality care compared to those in the control group [11]. On the other hand, a Cochrane systematic review [12] and a literature review found that several dimensions of PBF have not yet been adequately studied (e.g., impact on equity, organizational change, stakeholder satisfaction) [8,13]. The difficulties associated with the PBF program in Uganda demonstrated the importance of considering the context and the implementation process [14]. These difficulties included the lack of consideration of other health programs implemented simultaneously, insufficient communication between stakeholders, overly rapid selection of performance indicators, inability to modify inappropriate indicators over time, implementation delays causing institutional memory loss, limited ability of auditors to identify and tally contract-relevant services, overwhelming workload of extracting performance data, insufficient feedback meetings, and financial shortfalls [14].

Researchers have suggested that several dimensions must be taken into account when evaluating any PBF intervention, including the characteristics of the healthcare system. Most studies on PBF focus on primary healthcare facilities. Local stakeholders have highlighted the need to examine the implementation process of PBF in higher level health facilities. Other elements to be considered include the governance structure, organizational cultures, training activities, nature of contracts, redistribution of incentives, indicators of performance, mechanisms to monitor performance, means of dialogue between stakeholders, power relations, community involvement, and perceptions of stakeholders [8,15–17].

In addition, one must consider that PBF can also trigger unintended processes and effects. Financial incentives might erode intrinsic motivation [18] or lead to the neglect of non-incentivized services, as observed in Latin America and Rwanda [19,20]. Other risks include the migration of health personnel to better performing health centers, provision of healthcare to easily accessible populations, a decrease in the quality of care, a reduction in transfers of patients to other health facilities, and manipulating data regarding performance (gaming) [15].

### Combining supply- and demand-side interventions

Achieving universal health coverage will likely require a comprehensive approach that simultaneously tackles supply-side and demand-side barriers to effective healthcare coverage. Offering financial incentives to motivate healthcare providers may not be sufficient to tackle all the barriers that influence access to healthcare (e.g., user fees). Without making specific provisions for the poor, PBF is unlikely to produce the same benefits for all. To promote equitable access to healthcare, PBF could be combined with pro-poor targeting strategies and/or community-based health insurance (CBHI). Thus far, empirical evidence on the feasibility of combining PBF with other supply- or demand-side interventions that address social protection is scarce.

In LMICs, pro-poor targeting has been implemented both on the supply side and the demand side. On the supply side, one strategy is to pay healthcare providers more for services offered to the poor compared to the rest of the population [21]. On the demand side, one pro-poor targeting strategy is to exempt the worst-off from paying user fees. One challenge is the identification of the poorest. Recent studies have shown that community members can use selection procedures to identify the poor [22–25]. Such participatory processes to identify the poor has been found to be socially valued and effective in rural areas, but less so in urban areas. The challenges of pro-poor targeting strategies include conflicts of interests of management committees, lack of knowledge about policies, inability to travel in order to benefit from the services, apprehensions of healthcare providers, and diminished quality of care [26,27]. Rigorous studies on pro-poor targeting strategies are very rare in Africa, resulting in a knowledge gap on their implementation processes [26]. Thus far, no intervention or research program combined PBF with a community-based selection of the poor.

On the demand side, CBHI could be combined with PBF to increase risk sharing among the population. Since the 1990s [28], several experiments in sub-Saharan Africa have shown that CBHI membership facilitates access to healthcare [29–31] and offers a degree of financial protection [32–35]. However, participation in CBHI generally remains very low (10%–15% penetration rate [27]). Access to CBHI is often not equitable because the poorest populations do not have the financial capacity to pay membership fees [36–38]. Even when they become members, the poor are generally unable to benefit from healthcare coverage to the same extent as others due to indirect costs or copayments [39,40]. Moreover, healthcare providers often perceive the implementation of CBHI as a threat to their personal interests [41–44], which has important repercussions on the quality of services provided [45,46].

Although CBHIs have adopted a wide range of initiatives involving various stakeholders, none of them have been able to resolve the issue of low membership on their own [47]. To date, however, there have been no studies on the combination of PBF with CBHI.

It is essential to produce evidence on the feasibility of implementing combined interventions with synergetic potential, as a means to address equity concerns related to traditional PBF approaches. In Burkina Faso, the implementation of various intervention arms combining PBF with CBHI and pro-poor targeting offers a unique opportunity to explore existing gaps in knowledge.

### Study setting

In Burkina Faso, nearly half of the population lives below the poverty line and more than 100,000 children under the age of 5 die each year [48]. The probability that a newborn baby will die before reaching the age of 5 is 102 per 1,000 [2]. The maternal mortality ratio is 400 per 100,000 live births [3]. In the poorest quintile, only 12% of women receive prenatal care at least four times during their pregnancy, compared to 32% of women in the richest quintile [49]. The percentage of births attended by a skilled healthcare provider also ranges widely from 19% in the poorest quintile to 84% in the richest quintile [49]. With such important inequalities in access to care [50,51], the health of the poorest population is a concern.

In 2011, the Government of Burkina Faso launched a pilot PBF intervention targeting maternal and child healthcare services in three districts. In 2012, the evaluation of the pilot project [52] found a substantial improvement in the quantity and quality of healthcare services provided [52]. However, evaluators were not able to establish a causal relationship between PBF and the outcome variables due to the study design. With regard to the implementation process, results highlight the importance of ensuring that stakeholders are well informed, feel involved in the process, and accept the rules relating to incentive sharing. However, given the small scale of this evaluation, it remains unclear how the different elements of the PBF intervention, the context, and the stakeholders involved influenced each other to affect the intervention's effectiveness.

In 2013, the government decided to expand PBF to 12 districts in six regions, covering a population of approximately four million people. The operation is financed by the World Bank and carried out by the Ministry of Health's Technical Support Unit in charge of PBF (TSU- PBF). The objective remains that of improving the utilization and quality of maternal and child healthcare services. In comparison to the earlier pilot project, PBF will be combined with additional interventions directly targeting health equity: demand- and supply- side pro-poor targeting and CBHI. The objective of combining PBF with pro-poor targeting and CBHI is to ensure that healthcare improvements are accompanied by adequate measures to enhance equity in utilization rates and secure financial protection. Four intervention arms are being implemented to compare their outcomes:

- *Performance-based financing only* (PBF 1): Health facilities are paid according to the quantity and quality of healthcare services delivered. Patients are required to pay user fees. PBF performance agreements concluded between the MOH and the health center define the package of basic services to be provided and the indicators and targets to be reached in delivering these services. The results achieved against these targets are assessed by external reviewers every 3 months. Based on these verified results, each facility under a PBF contract receives payments in partial reimbursement for the services delivered. The payments are based on unit prices, based on a number of factors designed to achieve the desired results. This includes the basic cost of the inputs required (and not financed elsewhere) for services to be rendered, adjusted for quality of the service. This intervention group does not include any systematic targeting and subsidization of the poor.
- *Performance-based financing + systematic targeting and subsidization for the poor* (PBF 2): PBF is combined with a community-based selection of the poor (approximately 15%–20% of the population) who are entitled to benefit from user fee exemptions. A specific PBF indicator is introduced for purchasing services for the poor, using a fixed unit price for each consultation package (including services and drugs). The fixed unit price is higher than for non-targeted patients, taking into consideration the delivery of care without direct payments from patients. If issues of oversupply arise after the first few months of implementation, a cap may be established so that a maximum of about 10% of consultations can be reimbursed as consultations offered to the poor. In that case, health facilities would receive the normal rate for consultations offered to the poor exceeding the 10% cap.
- *Performance-based financing + systematic targeting and subsidization for the poor + provider motivation* (PBF 3): PBF is combined with a community-based selection of the poor who are entitled to benefit from user fee exemptions (approximately 15%–20% of the population). Healthcare providers are paid more for services provided to the poor than in PBF 2. The multiplication factor varies depending on the services. If issues of oversupply arise, a reimbursement cap of about 10% may be established. The higher reimbursement rate acts as a financial incentive for healthcare providers to take personal initiative to increase the utilization of healthcare by the poorest households.
- *Performance-based financing + community-based health insurance (including targeting of the poor)* (PBF 4): PBF is combined with CBHI offered to the general population. The insurance premiums of the poor (approximately 15%–20% of the population) are subsidized. Healthcare services are purchased at varying payment levels, as defined in PBF 3 (with higher payment levels for services provided to the poor). Thus, subsidization of services provided to the poor who do not pay the premiums will pass via the PBF mechanism at budget-neutral levels, while capitation payments will replace out-of-pocket payments for the general population.

Primary healthcare facilities are randomly allocated to the different groups. All district and regional hospitals included will be allocated to PBF 2.

**Figure 1:**
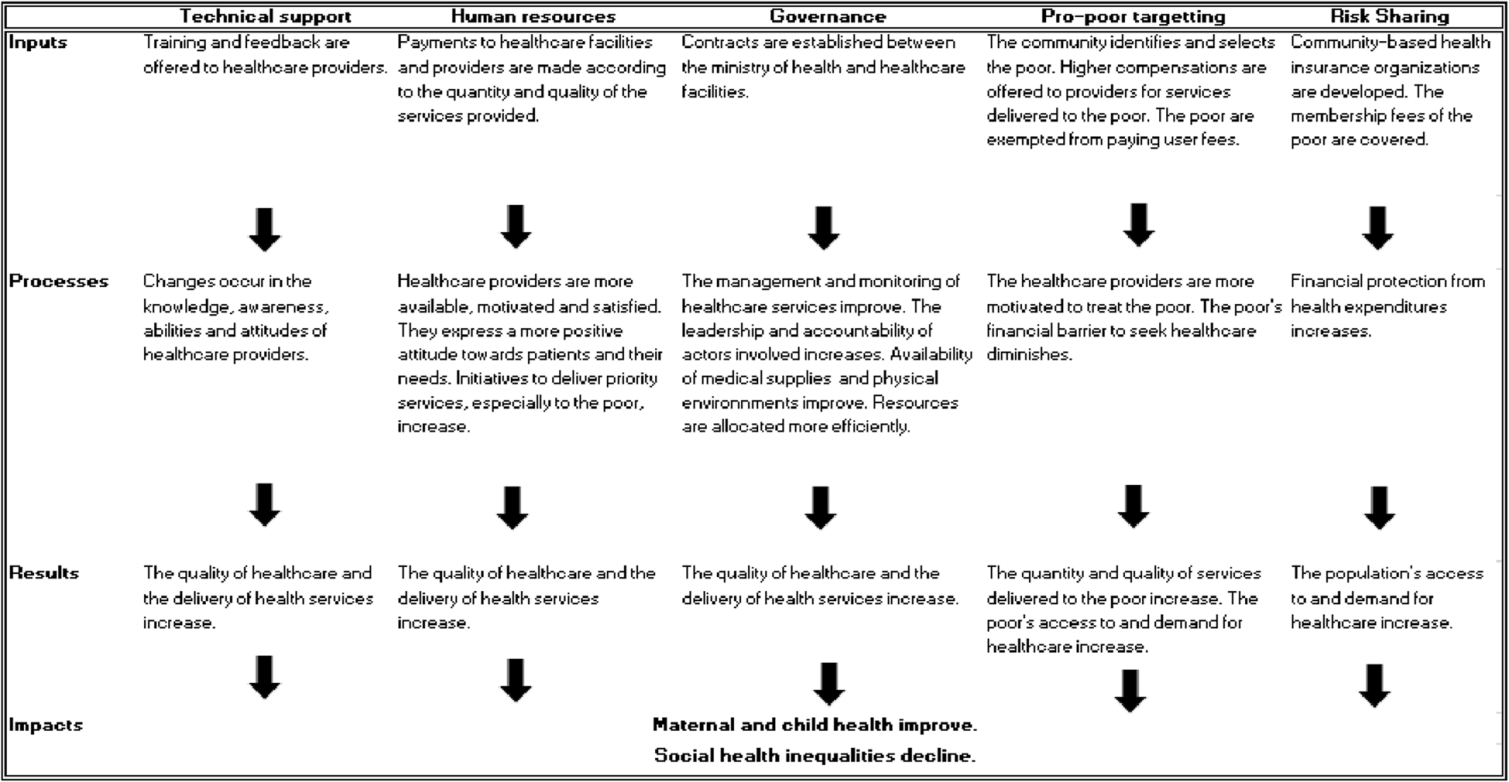
**The theory of intervention.**

The theory of the overall intervention combining PBF with pro-poor targeting and CBHI is presented in Figure 1. As with any real-life intervention, these four modalities (PBF 1, 2, 3, and 4) may be modified or adapted in the future. Four of the six regions implement PBF 1, PBF 2, and PBF 3; one region implements PBF 1 and PBF 4; and one region only implements PBF 1.

The World Bank also finances the impact evaluation of the four intervention arms. The impact assessment adopts a blended experimental and quasi-experimental design. For the experimental portion of the design, facilities within PBF districts are randomly assigned to PBF intervention arms to test the effects of the additional components (pro-poor targeting - PBF 2 and 3 and CBHI - PBF 4) versus PBF alone (PBF 1). For the quasi-experimental portion, facilities are matched with other facilities in neighboring control districts to compare the effects of the intervention arms with the absence of any intervention (counterfactual). Two comparison districts in each of the six regions, where the intervention districts will take place, have been selected to act as comparison health facilities. The research protocol presented in this manuscript is not for this impact evaluation. Our protocol focuses exclusively on the implementation process and, as such, adopts a design that is complementary to the one used for the impact evaluation.

### General objectives and research questions

The overall objective of this study is to analyze the emergence, the fidelity, the implementation process, the sustainability, the possibilities for scaling-up the intervention arms nationwide, and the ethical implications of the intervention arms. The research questions (Q) are:

- Q1. How was the decision taken to combine PBF with CBHI and pro-poor targeting?
- Q2. Were the interventions' activities implemented as planned and why?
- Q3. How do social actors perceive the interventions?
- Q4. How were the interventions implemented?
- Q5. What adaptation/invention/innovation strategies did the interventions trigger?
- Q6. Did the interventions induce unintended effects? If so, which ones?
- Q7. How did the different contexts shape the way the interventions were deployed?
- Q8. Which specific activities or combination of activities produced changes in the performance of health centers? How were these changes produced over time?
- Q9. Will the implementation process of the interventions allow their sustainability?
- Q10. What is the potential for scaling-up the interventions nationwide?
- Q11. Which ethical considerations are associated with the interventions?

## Methods

### Evaluation approach

The current study is anchored in methodological pragmatism [53–55]. When evaluating public health interventions, researchers are faced with complex and dynamic objects that are composed of various intertwined actions. These objects are often characterized by non-linear relations, organized in specific contexts, and understood as open systems that encompass the researcher [56,57]. For this type of evaluative study [58], researchers must adopt a pragmatic position to answer research questions and provide recommendations to decision makers [59].

### Evaluability assessment

The methodological strategy was developed during a pre-evaluative phase [60,61] which took place between July and December 2013. Through literature reviews, workshops, and dialogue with stakeholders, the evaluability assessment phase provided a better understanding of the interventions arms, their components, and their expected effects. We developed the research questions and methodological strategy based on the knowledge gaps, the theory of intervention [56], the available budget, policy-maker needs, and a realistic timeline [62].

### Types of evaluation

Several types of evaluations will be conducted as part of this study. Examples of typical questions representing each type of evaluation are presented in Table 1.

**Table 1:** **Examples of research questions for each evaluation type**

**Types of evaluation**	**Examples of research questions**
Emergence	- How was the problem of accessibility and performance recognized?
	- What were the debates surrounding the potential solutions?
	- What motivated the choice to select these intervention arms instead of others?
	- What were the positions of the different actors involved in the adoption of these intervention arms?
Performance	- Did the performance change in the different levels of health facilities? If so, why?
	- Were the performance objectives met since the beginning of the intervention arms?
	- Did the utilization rate of healthcare services change for the general population or vulnerable groups?
	- Did the quality of healthcare services change for the general population or vulnerable groups?
	- Did the level of motivation of health professionals change?
Fidelity	- Were the activities regarding the training of health professionals, the contracts, the monitoring, the payments, the user fee exemptions, and the CBHI implemented as initially planned?
	- Were any of these activities added, modified, or omitted?
Processes	- How do stakeholders perceive the implementation of the intervention arms?
	- Which factors facilitated or hindered the implementation of the intervention arms?
	- What are the unintended processes and effects caused by the intervention arms?
	- How did the intervention arms influence governance, management, monitoring, and leadership within the healthcare system?
	- How did the intervention arms influence interpersonal relations, communication, and collaboration between stakeholders?
	- How did the intervention arms influence the practices, behavior, and motivation of healthcare providers?
	- How did the intervention arms influence the available resources, medical supply, and the infrastructure?
	- How do the different components of the intervention arms interact?
Ethical considerations	- What are the ethical considerations related to the intervention arms?
	- How was the community involved in the implementation of the intervention arms?
	- How do stakeholders perceive the process of identification of the poorest that is conducted by the community?
Sustainability	- Which human, material, and financial resources necessary for the intervention arms' activities were integrated in the district's budget?
	- Were the intervention arms adequately adapted to the context? Are they compatible with local practice?
	- Is the implementation of the intervention arms in line with the priorities of the health authorities?
	- Is there a cultural relation between the intervention and the stakeholders (e.g., rituals, symbols)?
	- Did the interventions influence the healthcare system?
	- How were the rules and procedures institutionalized?
Scale-up	- How were the stakeholders that can contribute to a scale-up of the intervention arms implicated? Are they favorable and committed to a scale-up?
	- Do the intervention arms respond to a recurrent and persistent problem?
	- Do the contexts in which the intervention arms were tested reflect the rest of the country?
	- Could the human, material, and financial resources be mobilized to scale-up the intervention arms?

The assessment of *emergence* (Q1) will examine the factors that led to the decision to combine PBF with pro-poor targeting and CBHI. An analytical framework derived from the multiple streams approach will be used to understand public policy agenda setting [63].

Assessing the *fidelity* of implementation (Q2) is essential because stakeholders plan to implement a combination of interventions that they believe to be effective. But in order to be effective, the interventions must be either implemented as planned (to avoid type 3 errors [64]), or the changes/innovations brought about must be beneficial. The evaluation of fidelity compares the activities that were initially programmed with those that were implemented (content fidelity), as well as their level of achievement (fidelity of the temporal and geographical coverage) [65–67].

The assessment of *processes* (Q3–7) will focus on the internal dynamics of the intervention arms, the roles, perceptions and coping strategies of actors, the unintended effects, the evolution of actions, and the mediating effect of the context [68,69]. We also seek to understand local dynamics, the relationship between healthcare providers and the populations, the local history, and the socio-historical context that could affect the performance of primary healthcare centers (PHC) and district or regional hospitals, as well as other processes. We will also examine the adaptation processes, which may be essential to the interventions' effectiveness [66,68].

The *performance* assessment (Q8) will focus on activities related to the interventions in PHC and district or regional hospitals. It is related to an effectiveness analysis. Primary healthcare centers and hospitals will be considered to be effective when they are better than others (median) and they improve their score on indicators associated with the interventions' activities.

The *sustainability* assessment (Q9) [70] will focus on three types of events that help achieve different levels of sustainability [71,72]: i) specific to sustainability (stabilization of resources and organizational risk taking); ii) jointly related to sustainability and to the implementation of the interventions (incentives for actors, adaptation of activities, alignment of objectives, transparency of communication, shared cultural artifacts, integration of rules); and iii) specific to the implementation (sufficient resources and compatibility with the host institution).

The conditions to *potentially scale-up the interventions* (Q10) will be studied by examining the characteristics of the innovation (e.g., combining PBF with CBHI and pro-poor targeting), the context, the host organization (Ministry of Health), the implementation strategy, and the support team (TSU-PBF, World Bank) [73].

The assessment of *ethical considerations* (Q11) will involve analysis of the moral and political issues associated with the design and implementation of the intervention arms. The goal is to go beyond ethical considerations traditionally associated with procedural ethics in order to examine a wider scope of ethical implications pertinent to the interventions.

### Case studies

The design will be a longitudinal multiple case study with several nested levels of analyses [74–77].

### Selection of the context for case studies

Since the social context and the district teams' leadership affect the performance of the PHC in Burkina Faso [76,78,79], case studies will be conducted in 3 of the 12 districts. The sample size takes into account our resource constraints but is sufficient to represent a variety of contextual situations conducive to the process of analytical generalization [80]. Six of the 12 districts had to be removed from the potential case studies because they do not represent the normal context of the healthcare system. Given the constraints and the need to select districts in different regions, the three selected districts are Diébougou (in the Southwestern region), Ouahigouya (in the Northern region), and Solenzo (in the Boucle du Mouhoun region).

### Selection of contrasted cases

**Figure 2:**
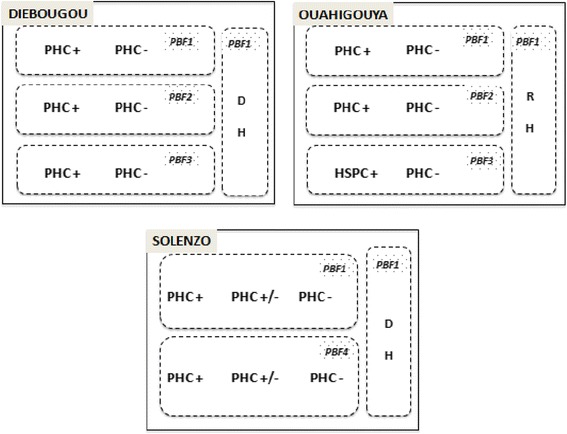
**Selection of cases within each district.**

The cases will be PHCs representing different intervention arms and the district or regional hospital located within each of the three selected districts. Figure 2 illustrates the selection of cases within each district. The total number of cases will be 18 PHCs (out of a potential 106 PHCs) and three hospitals (*n* =21 cases). More specifically, in Diébougou and Ouahigouya, where three modalities are implemented (PBF 1, PBF 2, and PBF 3), we will select two PHCs for each of the three intervention arms. In Diébougou, we will also select the district hospital (DH). In Ouahigouya, we will select the regional hospital (RH) because there is no district hospital. In Solenzo, where only two intervention arms have been implemented (PBF 1 and PBF 4), we will choose six PHCs, that is three for each of the two intervention arms, and the district hospital.

The selected cases will be considered to be typical cases [80]. For each intervention arm, we will choose the two or three most contrasted cases in terms of their initial performance (good, fair, poor) before the start of the interventions. We will define performance in relation to key activity indicators: number of births attended by a skilled healthcare provider, vaccination coverage, and number of preventive and curative consultations. We will assess the initial performance of each PHC over the 24 months preceding the intervention implementation. Based on a participatory approach with Health District Team member, we will select the PHC with the most contrasted performance scores using graphic reading and according to computed mean initial performance scores. The hypothesis of effectiveness and equity is the same for all PHCs, regardless of the initial performance. Thus, this case selection procedure follows the principal of “literal replication” whereby each case predicts similar results [80].

### Data collection

**Figure 3:**
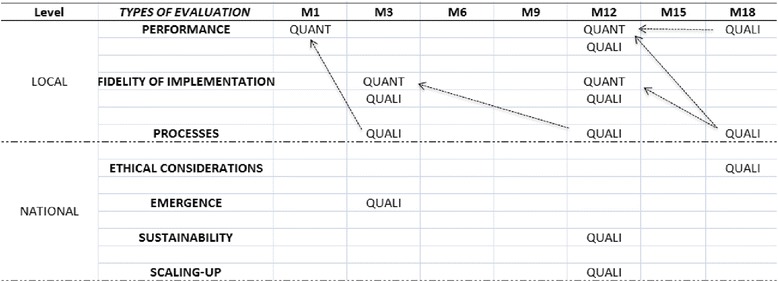
**Timeline for the quantitative and qualitative data collection.**

This evaluative research will adopt a mixed methods approach [81]. We will give more weight to the qualitative data collection (QUALI) compared to the quantitative data collection (QUANT). The temporality and the type of data collected for the different types of evaluations are presented in Figure 3.

During the first month (M1) of the study, we will measure the initial performance levels of the PHCs to allow the selection of cases. Our previous work in Burkina Faso and that of other researchers have demonstrated the quality of the routine data [76,77,82,83]. We will also organize a pilot case study in each district [80].

Three months (M3) after the beginning of the study, we will collect data to measure the fidelity of the intervention arms' implementation and to understand the process of implementation. Under the supervision of a researcher in each district, research assistants will be present in each case for a 2-week period. This relatively long immersion will not only create a sense of trust with the stakeholders, but also provide a better understanding of the contextual issues. On site, research assistants will conduct non-participant observation (e.g., at the health centers, in the villages). They will keep research diaries, take notes after informal interviews, and make ethnographic [84] summaries. The research assistants will begin formal data collection only during the second week in order to take into account the context and also the social dynamics by meeting important people. Research assistants will conduct in-depth interviews until we reach saturation of the data (at least 30 interviews) with key local stakeholders. This analysis (M3) will offer an opportunity to understand the performance of the PHC before the beginning of the interventions (Figure 3).

After 6 months (M6), a qualitative data collection round will be conducted on a national level to better understand the factors that contributed to the emergence of PBF with pro-poor targeting and CBHI. We will collect data from all of the available documentation and from approximately 20 in-depth interviews with key stakeholders. In-depth interviews will be conducted until we reach saturation of data.

After 12 months (M12), a second data collection phase will take place in all of the selected healthcare facilities. In terms of performance, quantitative data will be collected using the same method as in M1. However, without waiting for the results of these analyses, we will conduct in-depth interviews with healthcare providers and members of the management committee to capture their perceptions of the evolution of this performance. Following the same method used during M3, we will analyze the evolution of the fidelity of the implementation and the processes using document analysis, observations, and in-depth interviews. In addition, the sustainability and conditions to potentially scale-up the intervention arms nationwide will be studied through interviews conducted on a national level. We will conduct interviews until we reach saturation of data (approximately 30 interviews) with stakeholders.

At 18 months (M18), after analyzing the quantitative data on the evolution of the performance, research assistants will conduct a final 2-week field visit in each case. They will conduct interviews with key stakeholders to discuss and understand the quantitative performance data. Not only will we try to understand how the interventions are organized at M18 but, due to preliminary data analysis of M3 and M12, we will also conduct interviews on the evolution of the implementation and potential explanations for the challenges, modifications, and difficulties. We will also conduct the assessment of ethical considerations during this phase.

Each case has a specific historical, social, environmental, and political context as well as relations between the populations and healthcare providers. The temporal dimension of these relations is essential to understand, as the intervention arms will certainly bring about changes that will evolve over time [74,77]. The method adopted to collect data should be able to face this challenge.

### Analysis of case studies

**Figure 4:**
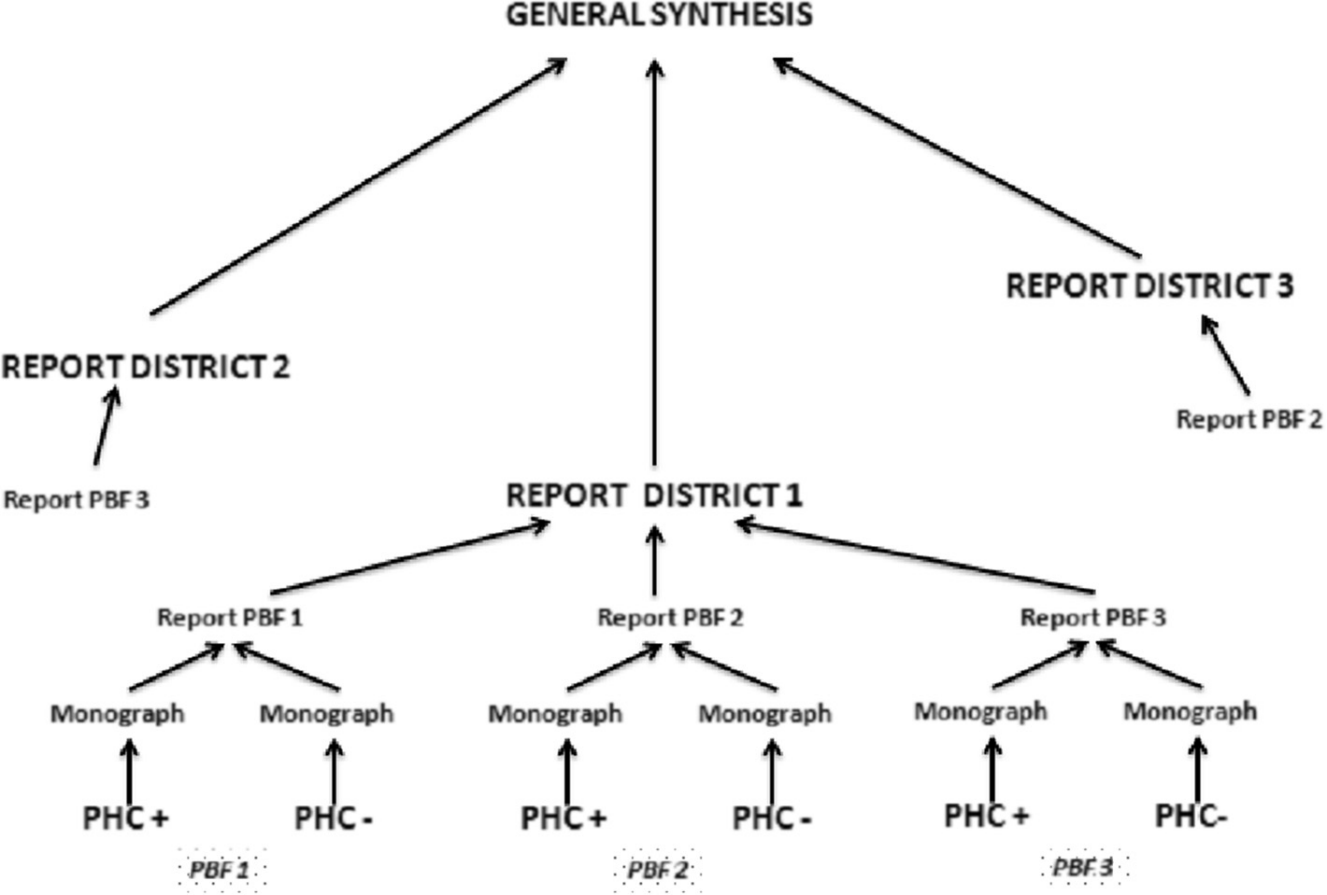
**Description of the general synthesis of results.**

The in-depth research strategy and use of multiple sources of evidence [76] reinforce the internal validity of the 21 case studies. We will organize the inter-case analyses as a stepwise process. After their field trips, each assistant will prepare a monograph of each case and a cross-sectional analysis of the intervention arm. We will conduct a quantitative analysis of the change in the performance of health centers by comparing the mean performance from the 24 months preceding the intervention with the mean performance obtained 1 year after the intervention. Then, each researcher will conduct a synthesis of the seven cases in the district. Finally, the principal investigator will analyze the 21 cases globally. For each of these steps, researchers will be able to move to a higher level of analytical generalization [82]. This collective process, illustrated in Figure 4, will strengthen the validity of the conclusions.

A preparatory workshop between researchers (M1) will ensure a common understanding of concepts, analytical framework, and data collection strategy. An expert will give a workshop on the NVivo software to the research staff in order to establish a common platform for the data analyses throughout the study. We will record, transcribe, and code all interviews. We will conduct content analyses [83,85]. We will organize collective analyses (workshop at M6 and M15).

### Ethics

There are no constraints or restrictions weighing on the autonomy and independence of the study or the publication of its results. Ethical committees in Burkina Faso and at the University of Montreal Hospital Research Center (CRCHUM) have approved the study. The study's objectives are aligned with Burkina Faso's national priorities. The Ministry of Health and donors have requested an evaluation of the four intervention arms. We will inform all relevant stakeholders and authorities about the purpose of the study and the presence of research assistants on the field. We will not disclose the performance of selected cases to the participants. We will obtain oral or written informed consent from the participants depending on their literacy level.

## Trial status

The study is financed through the first author's (VR) programmatic research funds allocated by the Canadian Institutes of Health Research (CIHR). The data collection will begin during summer 2014. The data cleaning or analyses will not have begun at the time of submission of this protocol.

## Discussion

An important strength of this protocol rests in its rigorous design. Langley et al. [86] have argued that process evaluations tend to adopt deceptively simple designs that rely on cross-sectional qualitative or ethnographic data. Many studies focus on only one level of analysis and fail to look at the evolution of the implementation process over time. For this study, we have adopted a longitudinal multiple case study with several nested levels of analysis. This study will collect real-time qualitative and quantitative data over a period of 18 months. We explicitly incorporated temporal progressions to help understand the intervention arms' complete life cycles, from their emergence to the conditions that may affect their sustainability. The prolonged involvement of researchers will allow them to be close to events and practices. Moreover, we will examine ongoing interactions among different groups of individuals and across multiple levels of healthcare provision. The study of PBF in higher level healthcare facilities is novel in Africa and responds to a knowledge gap. Overall, this complex study design will make a significant contribution to the field of health financing in LMICs as well as to the development of robust methodologies for process evaluations.

Moreover, the context in Burkina Faso provides a unique opportunity to examine the interactional implementation process of combined interventions. This study has been designed so that we can disentangle and compare the distinct contributions of PBF, pro-poor targeting, and CBHI. This will allow us to understand the added value of each component.

As with any population health intervention research, various methodological and operational challenges are likely to arise during the course of this study. One difficulty will be to avoid or control for the implementation of other studies or interventions overlapping with our area of interest. Health needs in Burkina Faso are urgent, so a number of prominent international health agencies are intervening with their own agendas or priorities at stake. The lack of coordination between all the actors makes it difficult to select cases with the assurance that no other confounding study or intervention will affect or contaminate our sample. Potentially confounding factors will be handled on a case-by-case basis, for example through negotiation with other stakeholders and adaptation of methodological strategies. Our close ties and constant communication with the country's Ministry of Health, funders, and local stakeholders will allow us to remain well informed throughout the study in order to take appropriate measures in a timely fashion, if needed.

Another limitation of this study will be the difficulty in establishing a causal chain between the inputs, the processes, and the results. Inferring causality for interventions implemented in a real-life setting is more complex, but on the positive side, results tend to be more pertinent for decision making. Another concern for this study is that multiple stakeholders have been directly implicated in the implementation of the interventions and may have vested interests in its promotion. Although the perceptions and strategies of the actors are part of the object of the present study, we must be wary of the possibility that participants may introduce biases in the study. Overall, we will try to strengthen the validity of the results by using multiple sources of data and varied methods of data collection and analysis.

This evaluation study will promote the utilization of the results. Throughout the study, the research team will collaborate with key decision makers and stakeholders to ensure that the research questions are pertinent and that the results will be useful for evidence-based decision making. This collaboration will flourish without influencing the scientific independence of research. We will organize workshops to plan the study and share results locally. We will disseminate the results using reports, policy briefs, and lay publications distributed in French and in English. These will also be shared on our webpage (www.equitesante.org). Moreover, we will present the results in local and international conferences. Lastly, we will publish scientific articles in open access journals to promote access to the findings.

## Conclusion

Independent evaluations of PBF in LMIC are rare and mainly examine impacts. To date, little research has helped to understand issues related to the implementation process and the coping strategies of actors that are at the heart of complex interventions. Moreover, it is urgent to clarify how PBF can be combined with other approaches to promote equitable access to healthcare services. The implementation of interventions with high synergistic potential in Burkina Faso provides a unique opportunity to respond to the existing knowledge gap. In this article, we have presented a comprehensive protocol that aims to study the emergence, the fidelity, the processes, the sustainability, the conditions that can influence scaling-up, and the ethical considerations of four intervention arms that combine PBF with pro-poor targeting and CBHI. The complex mixed methods design will allow researchers and decision makers to understand the evolution of these approaches over time. Ultimately, the scientific evidence produced should be used to promote access to high-quality healthcare for vulnerable groups.
